# The Role of *Dot1l* in Prenatal and Postnatal Murine Chondrocytes and Trabecular Bone

**DOI:** 10.1002/jbm4.10254

**Published:** 2019-12-17

**Authors:** Stephanie Y Jo, Miriam S Domowicz, Judith G Henry, Nancy B Schwartz

**Affiliations:** ^1^ Department of Radiology University of Pennsylvania Philadelphia PA USA; ^2^ Department of Radiology University of Chicago Chicago IL USA; ^3^ Department of Pediatrics University of Chicago Chicago IL USA

**Keywords:** EPIGENETICS, GENETIC ANIMAL MODELS, OSTEOARTHRITIS, OSTEOPOROSIS

## Abstract

Osteoarthritis and osteoporosis are widely prevalent and have far‐reaching public health implications. There is increasing evidence that epigenetics, in particular, histone 3 lysine 79 methyltransferase *DOT1L*, plays an important role in the cartilage and bone biology. In this study, we evaluated the role of *Dot1l* in the articular cartilage, growth plate, and trabecular bone utilizing conditional KO mouse models. We generated chondrocyte‐specific constitutive and inducible conditional *Dot1l* KO mouse lines using *Col2a1*‐*Cre* and *Acan‐CreER* systems. Prenatal deletion of *Dot1l* in mouse chondrocytes led to perinatal mortality, accelerated ossification, and dysregulation of *Col10a1* expression. Postnatal deletion of *Dot1l* in mouse chondrocytes resulted in trabecular bone loss decreased extracellular matrix production, and disruption of the growth plate. In addition, pharmacological inhibition of DOT1L in a progeria mouse model partially rescued the abnormal osseous phenotype. In conclusion, *Dot1l* is important in maintaining the growth plate, extracellular matrix production, and trabecular bone. © 2019 The Authors. *JBMR Plus* published by Wiley Periodicals, Inc. on behalf of American Society for Bone and Mineral Research.

## Introduction

Osteoarthritis is the most common arthritis affecting over 30 million people in the United States.^1^ The estimated direct medical cost of osteoarthritis is $185.5 billion per year^2^ and is expected to increase as the population ages and becomes more obese. With the exception of weight loss,^3^, ^4^ there is currently no convincing disease‐modifying therapy to halt the progression of osteoarthritis, and patients eventually need joint replacement. Although overall very safe and effective, up to 3% of patients require critical care services after elective arthroplasty.^5^ Additionally, complicated revision surgery may be necessary in up to 10%– to 12% of patients within 10 years of hip or knee arthroplasty.^6^, ^7^ These statistics are especially problematic for younger patients, who have longer life expectancies and also have higher activity levels.

Similarly, osteoporosis affects 5.1% of men and 24% of women over age 65 in the United States and is an important public health issue in the elderly.^8^ Resulting fractures, in particular hip fractures, can be devastating with up to 13% mortality in the first 30 days after surgery and up to 33% mortality within the first year after surgery.^9^ Although bisphosphonates, teriparatide, and denosumab are effective treatments, these medications do carry risks including osteonecrosis, atypical fractures, and in the case of denosumab, increased infection.^10^, ^11^

Recently, there is growing evidence that epigenetics has an important role in cartilage and bone biology. Disruptor of telomeric silencing 1 (Dot1) and its mammalian homolog DOT1L (DOT1‐Like) are a novel class of non‐redundant histone 3 lysine 79 (H3K79) methyltransferases.^12^, ^13^ It has been shown that human *Dot1l* polymorphism rs12982744 is associated with increased risk of osteoarthritis in European and Chinese populations in genome‐wide association studies.^14^, ^15^ *Dot1l* is expressed in the normal growth plate and articular cartilage of prenatal and skeletally mature mice,^16^ and may preserve cartilage health by preventing the hyperactivation of Wnt signaling,^17^ inhibiting osteoclastogenesis,^18^ and preventing age‐related and post‐traumatic osteoarthritis.^19^ This study further assesses the role of *Dot1l* in prenatal and postnatal chondrocytes and trabecular bone *in vivo* using conditional KO mouse models.

## Materials and Methods

### Chondrocyte‐specific *Dot1l* KO mouse line

All animal work was approved by the Institutional Animal Care and Use Committee at the University of Chicago (Chicago, IL, USA). Animals were housed in a standard animal facility maintained by the Animal Resource Center at the University of Chicago. A conditional *Dot1l* KO mouse line with loxp sites around the second exon was generated previously utilizing the Knockout Mouse Project repository.^20^

For evaluation of prenatal *Dot1l* deletion, the *Dot1l* KO mouse line was crossed with a *Col2a1‐Cre* mouse line^21^ until *Dot1l* KO allele homozygosity and *Col2a1‐Cre* heterozygosity (*Dot1l* ^*Δ/Δ*^; *Col2‐Cre*) were obtained. For control animals, littermates including Dot1l^Δ/Δ^, Dot1l^wt/Δ^, Dot1l^wt/Δ^; *Col2a1‐Cre* genotypes were used.

For evaluation of postnatal *Dot1l* deletion, the *Dot1l* conditional KO mouse line was crossed with the *Acan‐CreER* mouse line (Jackson Laboratory line *Agc1* ^*tm(IRES‐CreERT2)*^), which expresses tamoxifen‐inducible *CreER* ^*T2*^ under the *Acan* promoter^22^ until *Dot1l* KO homozygosity and *Acan‐CreER* heterozygosity (*Dot1l* ^*Δ/Δ*^; *Acan‐CreER*) were obtained. For control animals, the littermate *Dot1l* ^*Δ/Δ*^ genotype was used. Genotyping primers are listed in Supplemental Table S1.

### *Dot1l* deletion and evaluation of cell proliferation

Three‐week‐old weaning age mice with genotype *Dot1l* ^*Δ/Δ*^; *Acan‐CreER*, and gender‐matched littermate *Dot1l* ^*Δ/Δ*^ were used for *Dot1l*‐deletion experiments unless otherwise specified. *Dot1l* was deleted by intraperitoneal injection of two doses of 150 mg/kg of tamoxifen (Millipore‐Sigma T5648) dissolved in corn oil.^22^ Deletion of *Dot1l* was assessed with chondrocyte genomic DNA PCR and chondrocyte RNA qPCR of *Dot1l*.

For cell proliferation evaluation, *Dot1l* was deleted from 3‐week‐old mice for 4 weeks, and then intraperitoneally injected with 75 mg/kg of bromodeoxyuridine (BrdU). Mice were euthanized 48 hours after BrdU injection for tissue harvest.

### Chondrocyte harvest and genomic DNA and RNA extraction

Femoral head (4‐week‐old mice and 7‐week‐old mice) and xiphoid process (15‐week‐old mice) cartilage were dissected, placed in QuickExtract (Lucigen) for DNA or TRIzol (Invitrogen) for RNA, and homogenized. DNA or RNA was extracted per manufacturer instructions. DNA samples were used for genomic DNA PCR to confirm *Dot1l* second exon excision. RNA samples were reverse transcribed with the High Capacity cDNA Reverse Transcription Kit (Applied Biosystems) and used for qPCR of *Dot1l*, *Col2a1*, and *Acan* expression. qPCR was performed with iTaq Universal SYBR Green Supermix (Bio‐Rad). qPCR primers are listed in Supplemental Table S2.

### Whole‐mount Alcian Blue–Alizarin Red staining

Postnatal day 2 (P2) mice were fixed in 95% ethanol for 24 hours with agitation. Skin was removed and organs were eviscerated. The mice were placed in Alcian Blue 8GX (Millipore‐Sigma) cartilage staining solution for 1 week and dehydrated in 95% ethanol for 1 week. Soft tissues were cleared with 1% potassium hydroxide (KOH) solution for 3 to 4 days and placed in Alizarin Red (Millipore‐Sigma) bone‐staining solution for several days until staining was complete. Soft tissues were further cleared in graded KOH solutions. Samples were stored in glycerol–formaldehyde solution before acquiring images.

### mRNA *in situ* hybridization

P2 mice were prepared for *in situ* hybridization as described previously.^23^ Briefly, dissected limbs were sunk in 20% sucrose–10% formalin in PBS, embedded in gelatin, and cut on a sledge microtome. Sections were permeabilized and hybridized with digoxigenin‐ (DIG‐) labeled riboprobes, and DIG‐labeled RNA duplexes were detected with alkaline phosphatase/anti‐DIG Fab conjugate. Sense controls were always negative. The colorimetric reaction was carried out using 4‐nitro blue tetrazolium–5‐bromo‐4‐chloro‐3‐indolyl phosphate (NBT–BCIP; Roche Molecular Diagnostics) as substrates.

### μCT image acquisition and image analysis

Knee joints of 15‐week‐old mice were harvested after 12 weeks of *Dot1l* deletion. Mouse knee joint trabecular bone architecture at the metaphysis was assessed using μCT (μCT50; Scanco Medical) at the Rush University MicroCT Core in Chicago (https://www.rushu.rush.edu/research/rush-core-laboratories/rush-microct-and-histology-core). Samples were scanned at 55 kVp and 145 μA, with a 500‐ms integration time and 6‐μm isotropic voxel size. 3D images of knee joint and distal femoral and proximal tibial quantitative trabecular and cortical bone morphology variables were obtained using associated manufacturer's software. Because of inherent differences between male and female mice morphology such as bone volume/total volume (BV/TV) and trabecular thickness (Tb.Th), representative data from male mice are shown.

### Histological evaluation

Knee joint tissue was harvested after mice were euthanized and fixed overnight in 4% paraformaldehyde. Tissues were subsequently washed and stored in 70% ethanol solution. After the samples were scanned with μCT, they were decalcified for 2 days at room temperature with Osteosoft (Millipore‐Sigma 101728). Tissues were embedded in paraffin and sectioned by the University of Chicago Histology Core (https://htrc.uchicago.edu/services.php?service=1).

H&E stain was performed by the University of Chicago Histology Core. For Safranin O staining, sections were deparaffinized, incubated in Safranin O solution for 5 min, and counterstained with hematoxylin. For immunohistochemistry, sections were incubated with primary antibodies for chondroitin sulfate 1–500 (monoclonal CS‐56; Millipore‐Sigma) or BrdU 1:100 (BD Biosciences) overnight and appropriate secondary antibodies. Negative control slide for BrdU was from P5 mouse femur without BrdU injection stained with BrdU antibody. Sections were developed with the DAB kit (Pierce).

Quantifications of articular cartilage and growth plate thickness and stain intensity were performed using ImageJ software (NIH, Bethesda, MD, USA; https://imagej.nih.gov/ij/). Length was measured on the image and adjusted according to a scale bar. The ratio of intensity density (IntDen) of the region of interest over background was reported as stain intensity.

### Statistical analysis

Results were expressed as the mean ± SD for bar graphs. Student's *t* test (two‐tailed, unpaired) was used for bar graphs and scatter plots. *p* < 0.05 was considered statistically significant per convention.

### DOT1L‐inhibitor‐treated *Zmpste24‐*deficient mice

Mice forelimb samples were generous gifts from Dr López‐Otín from Universidad de Oviedo, Spain. These samples were from 6‐week‐old *Zmpste24* KO mice intraperitoneally injected with EPZ‐5676 inhibitor (2.5 mg/kg body weight; Selleck Chemicals) or vehicle once per day, every day for 12 weeks. Age‐matched untreated WT mice served as control. There were two forelimbs per each group.

Once formalin‐fixed forelimb samples were received, they were washed in 70% ethanol and images were acquired with University of Chicago PaleoCT (http://luo-lab.uchicago.edu/paleoCT.html). Samples were scanned at a voltage of 70 kV, current of 115 μA, exposure time of 200 ms, and isotropic voxel size of 4.7 μm. Raw data were processed by datos|x software (GE Healthcare) reconstruction program using the Feldkamp algorithm. The projections underwent automatic geometric calibration, and were cropped and sliced. Resulting images were analyzed with ImageJ software using BoneJ plugin.

## Results

### Altered ossification in prenatal *Dot1l‐*deleted mice

To understand the role of *Dot1l* in the development of prenatal cartilage, we generated a *Dot1l* KO model under the control of the constitutive *Col2a1* promoter (*Dot1l* ^*Δ/Δ*^; *Col2‐Cre*). Interestingly, the number of live births of homozygous *Dot1l* KO mice was substantially lower than expected: Of the 181 animals born from 27 litters, only 8 animals were homozygous *Dot1l* KO, whereas the expected Mendelian number is 58. Furthermore, the pups needed to be genotyped early between P0‐2. Genotyping at conventional age P11‐14 revealed only one homozygous pup, which survived to adulthood, but displayed major growth retardation (data not shown). Genotype was verified using skin and tail‐tip samples (data not shown).

**Figure 1:**
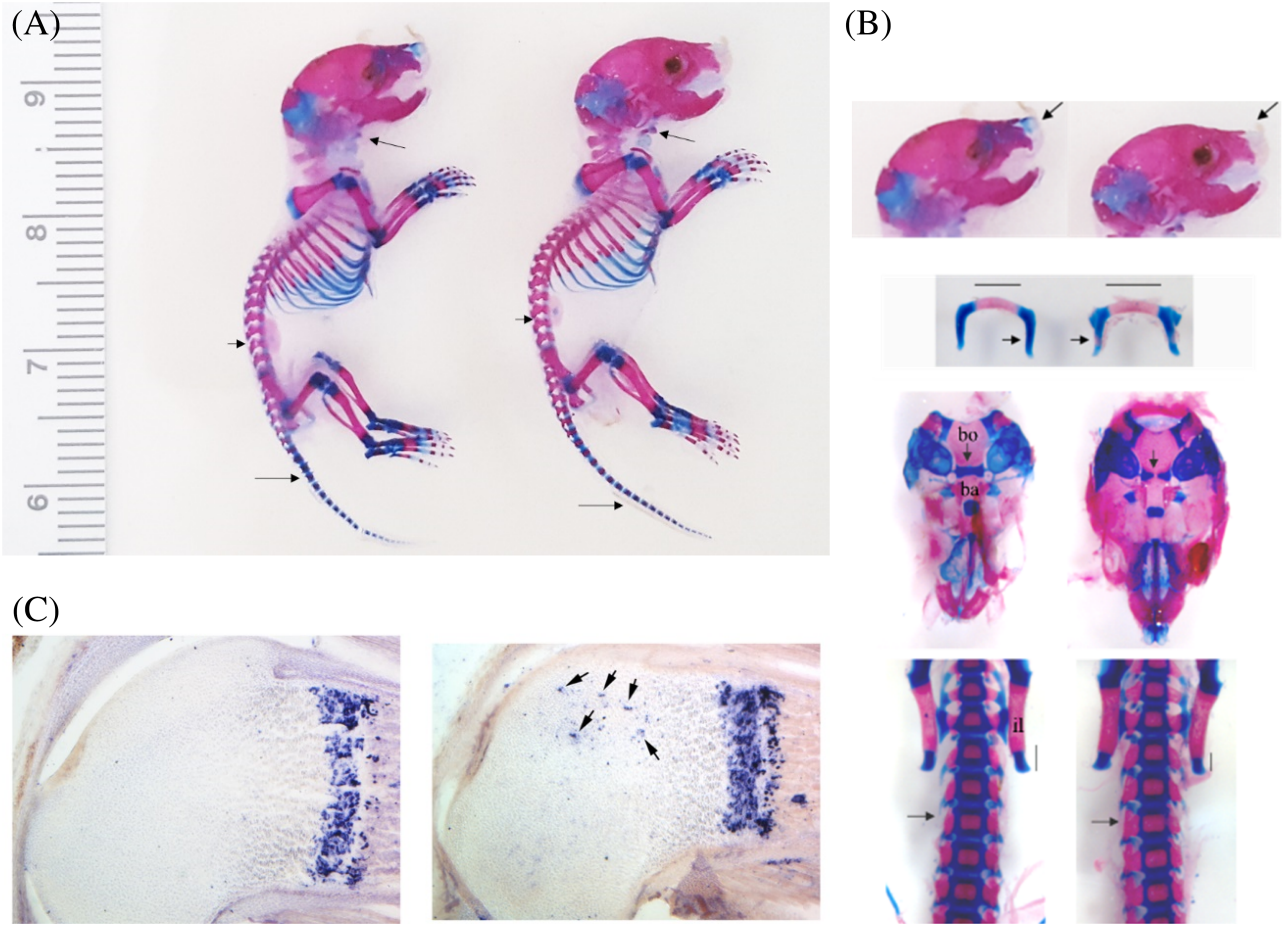
Whole‐mount Alcian Blue stain of P2 *Dot1l^wt/Δ^;Col2Cre* and *Dot1l* ^*Δ/Δ*^;*Col2Cre* mice (*n* = 2). (*A*) There is no gross difference in size between control littermate and *Dot1l*‐deleted mice. (*B*) Close‐up of dissected P2 Alcian Blue and Alizarin Red stained mice showed accelerated ossification of skull base, hyoid bone, and posterior elements of spine; arrows indicate areas of precocious mineralization. Bo = basioccipital bone; Ba = basisphenoid bone; il = iliac bone. (*C*) P2 mice RNA *in situ* hybridization with *Col10a1* probe showed abnormal expression in the epiphysis (arrows).

We performed whole‐animal Alcian Blue–Alizarin Red staining of the P2 homozygous *Dot1l* KO mice to investigate phenotypes that could account for the poor survival. Homozygous *Dot1l* KO pups did not exhibit gross difference in size (Fig. 1 *A*). However, with dissection, several elements showed accelerated ossification compared with control. Most notable accelerated ossification was observed in hyoid, skull base, iliac crest, and lumbar vertebrae elements (Fig. 1 *B*). The hyoid bone showed increased mineralization in the central section as well as incipient calcification of the long horn. At the base of the skull, after the mandible was removed to enhance the view of the cranial base, increased ossification was noticed between the basioccipital and the basisphenoid bone. The iliac crest was smaller, indicative of advanced ossification of the ilium. Similarly, the vertebrae elements of the lumbar spine showed advance mineralization as evidenced by larger Alizarin Red stain at the expense of Alcian Blue stain. In some cases, precocious mineralized vertebrae were observed between the ossified centrum and neural arch.

Given the accelerated ossification, we next evaluated if *Dot1l* affects hypertrophic chondrocyte differentiation and subsequent endochondral ossification. mRNA *in situ* hybridization of *Col10a1*, a marker of hypertrophic chondrocytes,^24^ was performed in the femur of P2 pups. Although there was comparable level of *Col10a1* expression in the *Dot1l*‐deleted and control growth plates, there was ectopic expression of *Col10a1* in epiphyseal chondrocytes (Fig. 1 *C*, arrows).

### μCT evaluation of knee joint in postnatal *Dot1l‐*deleted mice

Because of the difficulty obtaining sufficient number of animals for experimentation, we developed a chondrocyte‐specific inducible conditional *Dot1l* KO mouse line using *Acan‐CreER* system to evaluate the role of *Dot1l* in postnatal mice. Three‐week‐old weaning age mice were intraperitoneally injected with tamoxifen. After 4 weeks of tamoxifen treatment, a 50% reduction of *Dot1l* expression was observed by qPCR (Supplemental Fig. S1). Although there was no gross difference in the body size between the control and *Dot1l‐*deleted mice at 15 weeks of age, 12 weeks after *Dot1l* deletion (data not shown), there was mild decrease in body weight in *Dot1l‐*deleted mice (Supplemental Fig. S2).

**Figure 2:**
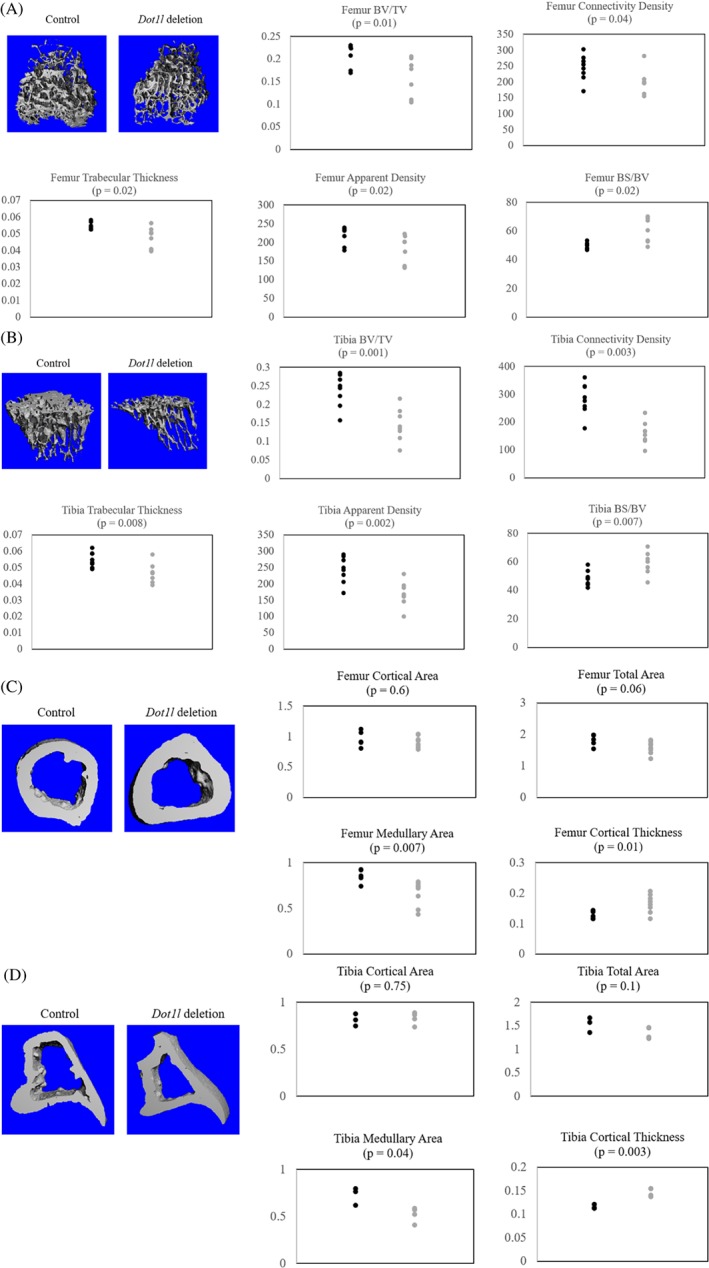
μCT analysis of *Dot1l* ^*Δ/Δ*^ and *Dot1l* ^*Δ/Δ*^; *Acan‐CreER* littermates at 15 weeks of age, 12 weeks after *Dot1l* deletion (*n* = 8). (*A*) Trabecular bone μCT 3D rendering and quantitative analysis of distal femur of control and *Dot1l*‐deleted mice showed decrease in trabecular bone. (*B*) Trabecular bone μCT 3D rendering and quantitative analysis of proximal tibia of control and Dot1l‐deleted mice showed decrease in trabecular bone. (*C*) Cortical bone μCT 3D rendering and quantitative analysis of distal femur of control and *Dot1l*‐deleted mice showed increase in cortical bone thickness and decrease in medullary area. (*D*) Cortical bone μCT 3D rendering and quantitative analysis of proximal tibia of control and *Dot1l‐*deleted mice showed increase in cortical bone thickness and decrease in medullary area. Black = control; gray = *Dot1l* deletion; BV/TV = bone volume/total volume; BS/BV = bone surface/ bone volume.

For more detailed evaluation, mouse knee joints were harvested and μCT was performed. With *Dot1l* deletion, there was qualitative and quantitative decrease of trabecular bone in the distal femur and proximal tibia (Fig. 2 *A*,*B*). Quantitative evaluation showed statistically significant decreases in BV/TV, Tb.Th, connectivity density, and apparent density of trabecular bone in the *Dot1l‐*deleted mice compared with age‐matched controls. There was also increased BS/BV consistent with decreased Tb.Th.^25^ The decreased apparent density may account for the mild decreased body weight in *Dot1l‐*deleted mice without a gross difference in size. Next, we examined the cortical bone at the metaphysis. Notably, there was decreased medullary space and increased cortical thickness of femur and tibia both qualitatively and quantitatively (Fig. 2 *C*,*D*). These findings suggest that *Dot1l* loss in chondrocytes led to weakened trabecular bone with likely adaptive response of the cortical bone.

### Reduction of extracellular matrix in articular cartilage and growth plate in postnatal *Dot1l‐*deleted mice

**Figure 3:**
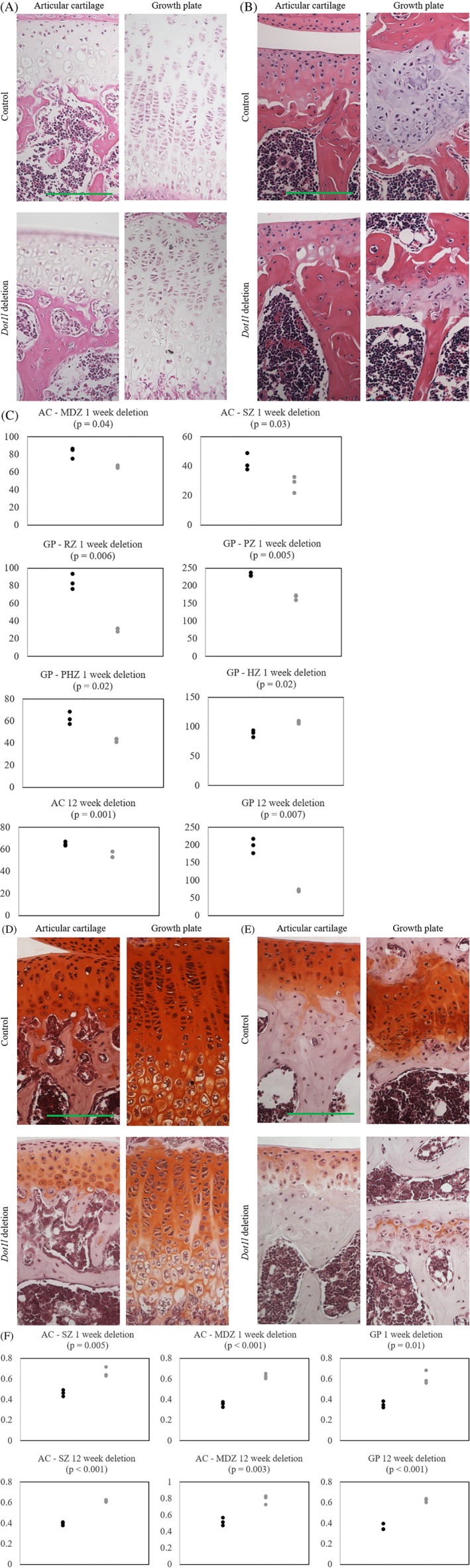
H&E stain and Safranin O stain of knee joint in *Dot1l* ^*Δ/Δ*^ and *Dot1l* ^*Δ/Δ*^; *Acan‐CreER* littermates. (*A*) H&E stain of knee joints of control and *Dot1l*‐deleted mice at 4 weeks of age, 1 week after *Dot1l* deletion (*n* = 3). There was disorganization of the growth plate hypertrophic zone and an overall shortening of the growth plate. There was mild decreased articular cartilage thickness without fibrillation or cleft. (*B*) H&E stain of knee joints of control and *Dot1l*‐deleted mice at 15 weeks of age, 12 weeks after *Dot1l* deletion (*n* = 4). There was persistent marked decrease in articular cartilage and growth plate thickness. (*C*) Quantification of articular cartilage and growth plate lengths of control and *Dot1l*‐deleted mice in Fig. 3 *A* and *B*. Safranin O stain of knee joints of control and *Dot1l*‐deleted mice at (*D*) 4 weeks of age, 1 week after *Dot1l* deletion, and (*E*) 15 weeks of age, 12 weeks after *Dot1l* deletion. There was marked decrease in extracellular matrix with *Dot1l* deletion. (*F*) Quantification of Safranin O stain intensity of control and *Dot1l*‐deleted mice in Fig. 3 *D* and *E*. Black = control; gray = *Dot1l* deletion; AC = articular cartilage; SZ = superficial zone; MDZ = middle‐deep zone; GP = growth plate; RZ = resting zone; PZ = proliferative zone; PHZ = prehypertrophic zone; HZ = hypertrophic zone. Scale bar = 100 μm.

H&E stain of mouse knee joint growth plate and articular cartilage was performed in 4‐week‐old mice, 1 week after *Dot1l* deletion in *Dot1l* ^*Δ/Δ*^ and *Dot1l* ^*Δ/Δ*^; *Acan‐CreER* mice (Fig. 3 *A*). Growth plate showed decreased length with irregular cell stacking of the hypertrophic zone, shortening of the proliferative zone, and slightly enlarged hypertrophic cells. On the other hand, articular cartilage showed mild decreased articular cartilage thickness without surface fibrillation or cleft. We next evaluated the growth plate and articular cartilage in 15‐week‐old mice after 12 weeks of *Dot1l* deletion (Fig. 3 *B*). More marked shortening of the articular cartilage and growth plate were observed, but again no articular cartilage damage was apparent. Quantification of articular cartilage and growth plate lengths noted in Fig. 3 *C*.

To establish whether these changes were in part related to reduction in extracellular matrix deposition, Safranin O staining of the same samples was performed. Reduced Safranin O staining indicating loss of extracellular matrix in articular cartilage and growth plate was observed as early as 1 week after *Dot1l* deletion (Fig. 3 *D*). Particularly, there was loss of matrix deposition in the articular cartilage superficial zone and growth plate hypertrophic zone. Reduction of matrix deposition in the articular cartilage and growth plate became even more extensive after 12 weeks of *Dot1l* deletion (Fig. 3 *E*). Quantification of stain intensity noted in Fig. 3 *F*.

Because we observed increased ossification of the lumbar spine with prenatal *Dot1l* deletion, we also examined L3/4 discs 1 week and 12 weeks after postnatal *Dot1l* deletion using H&E and Alcian Blue stains. We observed similar hypertrophic zone disruption and decreased extracellular matrix deposition in vertebral discs of *Dot1l‐*deleted mice (Supplemental Fig. S3). Although these findings indicated that *Dot1l* is essential in maintaining extracellular matrix production and growth plate differentiation, *Dot1l* loss alone did not result in rapid articular cartilage damage.

**Figure 4:**
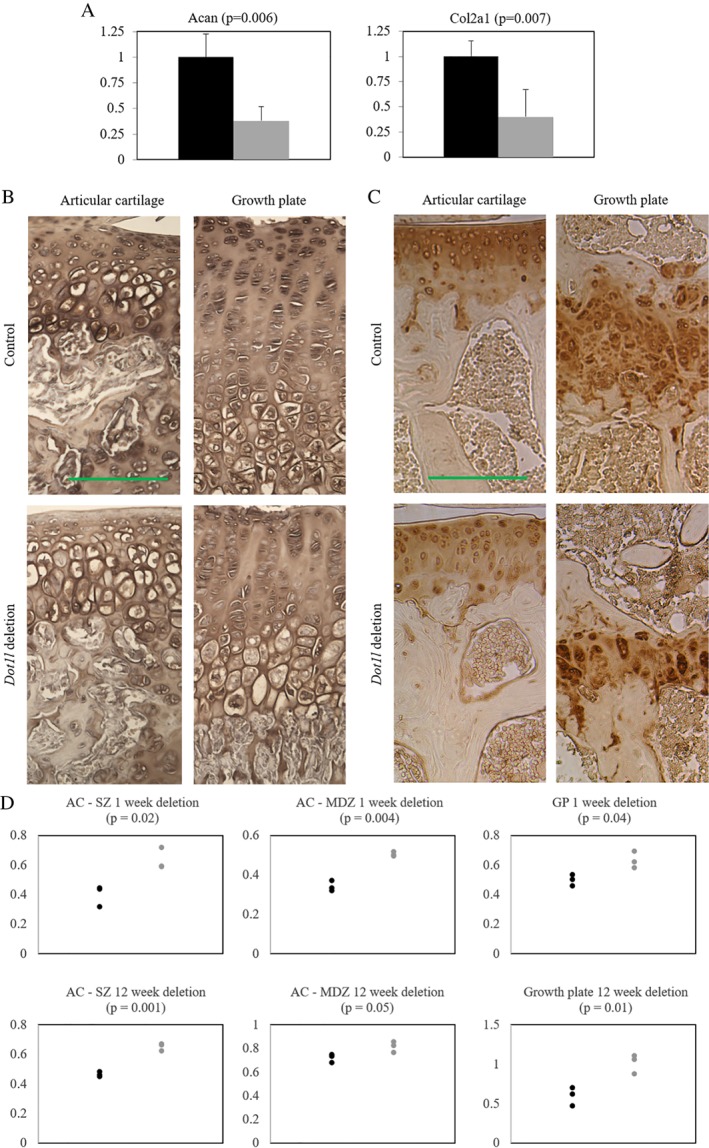
Decreased extracellular matrix production with *Dot1l* deletion in *Dot1l* ^*Δ/Δ*^; *Acan‐CreER* mice. (*A*) mRNA expression of *Acan* and *Col2a1* in chondrocytes was decreased with *Dot1l* deletion in *Dot1l* ^*Δ/Δ*^; *Acan‐CreER* mice at 4 weeks of age, after 1 week of deletion (*n* = 3). Black = control; gray = *Dot1l* deletion. Chondroitin sulfate immunohistochemical stain of mouse knee joint at (*B*) 4 weeks of age, 1 week after *Dot1l* deletion (*n* = 3), and (*C*) 15 weeks of age, 12 weeks after Dot1l deletion (*n* = 4) showed marked decrease in chondroitin sulfate, particularly the articular cartilage SZ. (*D*) Quantification of chondroitin sulfate signal intensity. Black = control; gray = *Dot1l* deletion; AC = articular cartilage; SZ = superficial zone; MDZ = middle‐deep zone; GP = growth plate. Scale bar = 100 μm.

Next, we performed *Acan* and *Col2a1* qPCR of 4‐week‐old *Dot1l* ^*Δ/Δ*^ and *Dot1l* ^*Δ/Δ*^; *Acan‐CreER* mice femoral head chondrocytes 1 week after *Dot1l* deletion. There was 62% and 60% reduction in *Acan* and *Col2a1* expression, respectively (Fig. 4 *A*), which correlated with the reduced Safranin O staining. There was also decreased chondroitin sulfate expression, determined by immunohistochemistry with the CS‐56 antibody 1 week (Fig. 4 *B*) and 12 weeks (Fig. 4C) after *Dot1l* deletion, reflecting reduced expression of the ACAN core protein. Quantification of stain intensity noted in Fig. 4D. These results confirm that *Dot1l* is an important modulator of the proteoglycan component of the extracellular matrix.

### Decreased cell proliferation in postnatal *Dot1l‐*deleted mice

**Figure 5:**
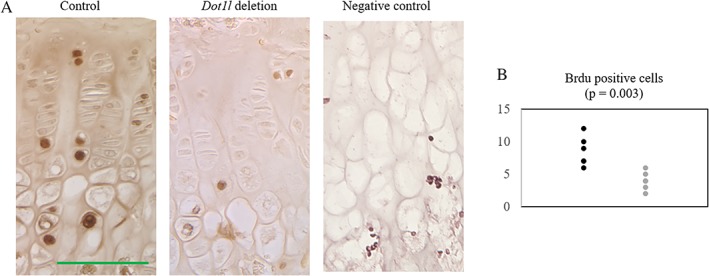
BrdU‐positive cells were decreased with *Dot1l* deletion in *Dot1l* ^*Δ/Δ*^; *Acan‐CreER* mice after 48 hours (control, *n* = 3; experiment, *n* = 4). (*A*) BrdU staining of mouse knee joints in 7‐week‐old mice, 4 weeks after *Dot1l* deletion showed decrease in BrdU‐positive cells in the *Dot1l*‐deleted growth plate. Control slide is from P5 mouse femur, not injected with BrdU. (*B*) Quantification of BrdU‐positive cells per 0.01μm^2^. Cells were counted in two different regions per slide. Black = control; gray = *Dot1l* deletion. Scale bar = 100 μm.

Dot1/DOT1L is a crucial regulator of the cell cycle and loss of Dot1/DOT1L leads to impaired cell division.^26^, ^27^, ^28^ Beacuse shortening of the proliferative zone was observed in growth plates of *Dot1l* deletion in *Dot1l* ^*Δ/Δ*^; *Acan‐CreER* mice (Fig. 3 *A*,*B*), we evaluated whether *Dot1l* deletion led to altered proliferation of chondrocytes. Seven‐week‐old *Dot1l* ^*Δ/Δ*^ and *Dot1l* ^*Δ/Δ*^; *Acan‐CreER* mice (4 weeks after *Dot1l* deletion) were injected with BrdU and tissues were harvested after 48 hours. Detection with a BrdU antibody showed decrease in BrdU‐positive cells in *Dot1l‐*deleted growth plate (Fig. 5), corroborating the shortened growth plate observed with H&E staining after *Dot1l* deletion. Immunohistochemistry with cleaved Caspase 3 was performed on the same samples, but there was no appreciable difference between *Dot1l*‐deleted mice and control mice (data not shown).

### Phenotype of *Dot1l* loss in bone morphology is dependent on nuclear lamina integrity

**Figure 6:**
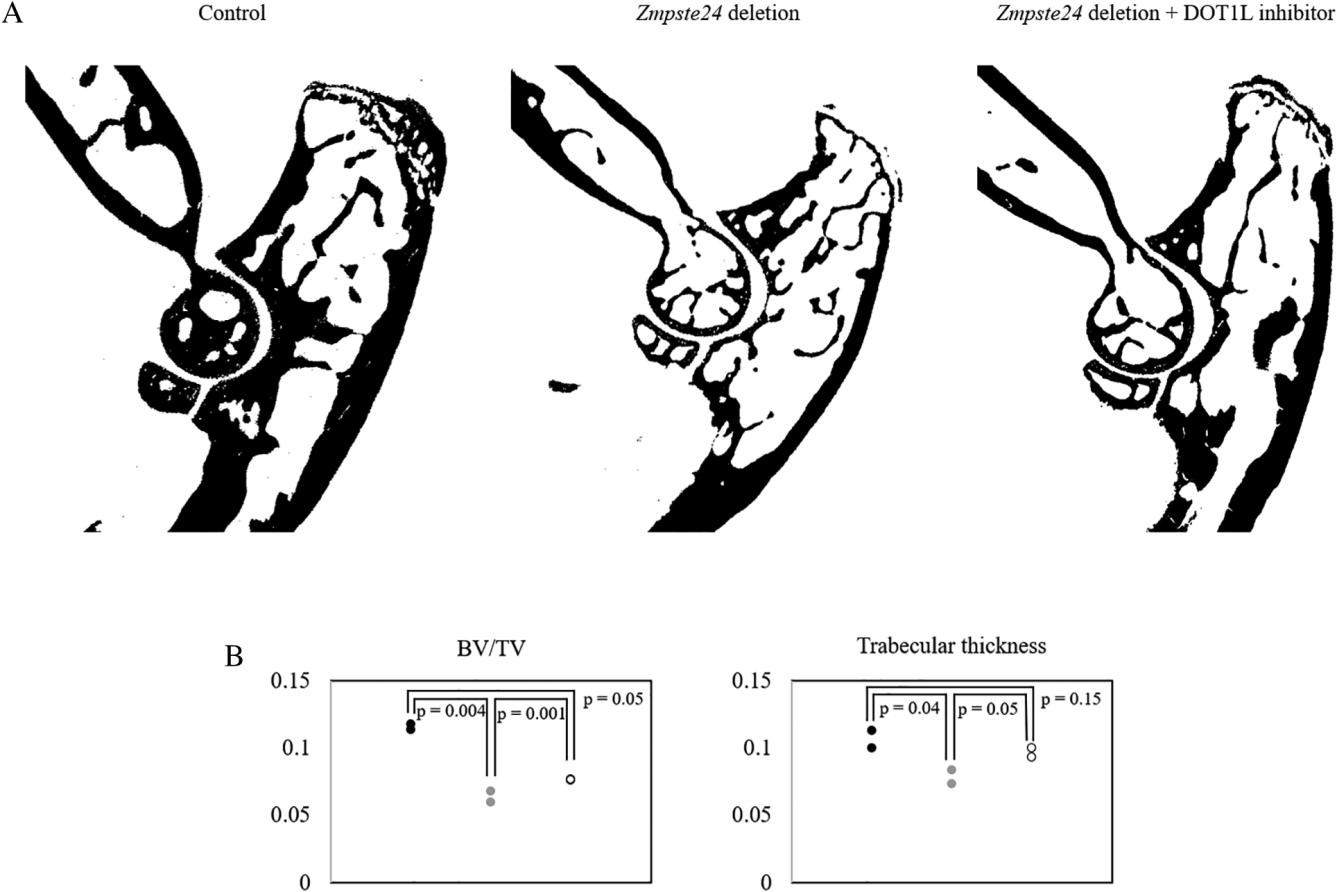
Three months of DOT1L inhibitor treatment of *Zmpste24*‐deficient mice partially rescued bone loss in the progeria model (*n* = 2). (*A*) 2D μCT images of mice forelimb showed qualitative decrease in trabecular bone in *Zmpste24*‐deleted mice compared with control, which is partially improved with DOT1L‐inhibitor treatment. (*B*) Quantitative analysis showed partial improvement in BV/TV and trabecular thickness in *Zmpste24*‐deleted mice treated with DOT1L inhibitor. Black = control; gray = *Zmpste24* deletion; white = *Zmpste24* deletion + DOT1L inhibitor.

As osteoarthritis and osteoporosis are strongly correlated with aging, we next turned to progeria model *Zmpste24‐*deleted mice. ZMPSTE24 is a prelamin‐processing zinc metalloproteinase and deficiency of this enzyme in humans results in premature aging syndrome.^29^ Previous studies have shown that in *Zmpste24*‐deleted mice, alterations in histone modification affects chromatin organization,^30^ and in particular, depletion of H3K9 methylation in these mice can rescue bone mineral density (BMD) loss.^31^ Based on these studies, we evaluated the effects of DOT1L inhibitor EPZ‐5676 in *Zmpste24*‐deleted mouse forelimbs using μCT. Forelimb samples of WT mice, *Zmpste24‐*deleted mice, and *Zmpste24‐*deleted mice treated with DOT1L inhibitor were gifts from Dr López‐Otín. Six‐week‐old *Zmpste24‐*deleted mice were treated with either vehicle or DOT1L inhibitor for 12 weeks. Age‐matched WT mice without treatment were used as reference. In *Zmpste24‐*deleted mice, there was marked decrease in BV/TV and Tb.Th, which was partially rescued in mice treated with the DOT1L inhibitor (Fig. 6). These results correlate with the existing evidence that alterations in histone modification can rescue bone loss in progeria models.

## Discussion

Our results showed that *Dot1l* is important in mouse prenatal and postnatal articular cartilage, growth plate, and trabecular bone. *Dot1l* deletion in chondrocytes during embryonic development led to embryonic and perinatal mortality, altered ossification, and dysregulation of *Col10a1* expression. Postnatal chondrocyte *Dot1l* deletion led to trabecular bone loss including decreased BV/TV, Tb.Th, connectivity density, and apparent density, and increased BS/BV. This was likely caused by disorganization of the growth plate hypertrophic zone with *Dot1l* deletion seen in histology. On the other hand, there was decreased medullary space and increased cortical bone thickness, likely compensatory. There was also decreased extracellular matrix production and corresponding decrease in *Acan*, *Col2a1*, and chondroitin sulfate expression in articular cartilage and the growth plate as early as 1 week after *Dot1l* deletion that persisted to 12 weeks. *Dot1l* deletion also led to decreased growth plate cell division, which resulted in shortening of the proliferative zone.

Previously reported cross of the conditional *Dot1l* KO line with the *Col2‐Cre* line was more successful in generating mice that survived *Dot1l* deletion,^17^ possibly because of decreased penetration of the deletion than in the present study; however, profound growth retardation at 1 month of age was reported. The previous study did not go into details on the number of pups born, but a different *Col2‐Cre* mouse line in B6SJL/F1 background was used, which possibly contributed to the difference in observed phenotype. In our study, a single *Dot1l* ^*Δ/Δ*^; *Col2‐Cre* mouse that survived to adulthood showed major growth retardation as well (data not shown), suggesting that the acceleration of ossification observed at P2 eventually extended to all elements. One possible explanation of the perinatal mortality is that early ossification of the hyoid bone and skull base had adverse effects on development or exerted mass effect on the trachea and brainstem, respectively. Although increased ectopic bone formation around the knee joint was noted in 16‐month‐old *Dot1l* heterozygote mouse,^19^ this likely reflects osteophyte formation from accelerated osteoarthritis and is unlikely to affect survival. Further studies are needed to delineate how prenatal *Dot1l* loss in *Col2a1‐*expressing cells effects survival, but it is important to point out that KO models of extracellular matrix components such as *Acan* 32, 33 and *Col2a1* 34 are also embryonic lethal. Furthermore, accelerated hypertrophy of chondrocytes was reported in the *Acan* KO mice.^32^, ^33^

We also observed ectopic expression of *Col10a1* in the P2 epiphysis with prenatal *Dot1l* deletion. As *Col10a1* expression reflects cell cycle exit, this result is congruent with decreased BrdU‐positive cells in growth plate seen with postnatal *Dot1l* deletion, and together suggest that *Dot1l* is required to maintain chondrocytes in the proliferative state. No previous studies have specifically examined cell cycle in *Dot1l‐*deleted growth plates. However, regulation of cell cycle and differentiation by *Dot1l* has been reported in other cell types, including hematopoietic cells^20^ and cortical neurons.^35^

*Dot1l* loss in postnatal mouse chondrocytes also led to weakened trabecular bone. In particular, there was decrease in total trabecular bone (BV/TV) as well as changes in bone morphology (BS/BV), both of which are correlated with callus strength of healing fracture.^36^ In contrast, there was increased cortical bone thickness and decreased medullary space, which may be compensatory. The trabecular bone loss was consistent with a recent study of DOT1L‐inhibitor treatment of ovariectomized mice,^18^ and increasing DOT1L activity may be therapeutic in human osteoporosis, particularly in postmenopausal women. The mechanism of bone morphologic change after *Dot1l* deletion in chondrocytes remains unknown. Altered growth plate integrity by reducing matrix deposition and proliferation could affect the bone formation by affecting the osteoblast, osteocyte, and osteoclast genesis. In fact, it has been reported that a proportion of hypertrophic chondrocytes can differentiate into osteoblast and osteocytes during endochondral bone formation and bone repair.^37^, ^38^ Involvement of *Dot1l* in this process remains to be explored. In addition, in human osteoarthritis, cartilage degenerative changes are associated with increased turnover in the adjoining bone, but reduced turnover in the nonadjoining bone,^39^ indicating interaction between cartilage integrity and surrounding bone.

Despite significant decrease in the extracellular matrix and thinning of the growth plate, we did not see fibrillation or cleft of the articular cartilage. This is likely related to the sedentary laboratory mice setting and a relatively short observation period (12 weeks of *Dot1l* deletion). A recent study showed that with an extended observation period (14 and 16 months) and under stress (surgically induced osteoarthritis model), *Dot1l* loss led to accelerated osteoarthritis in mice.^19^ These findings suggest that increasing DOT1L activity may be protective in human osteoarthritis.

We also observed partial rescue of the bone loss phenotype in *Zmpste24*‐deleted mice treated with the DOT1L inhibitor. This finding is consistent with H3K9 methylation depletion in *Zmpste24*‐deleted mouse leading to improved BMD,^31^ but it is somewhat contrary to the known histone methylation code. H3K9 methylation is associated with transcription suppression and chromatin silencing, whereas H3K79 methylation is associated with transcription activation and euchromatin.^26^, ^31^ The result is also somewhat divergent from our observation of trabecular bone loss with postnatal *Dot1l* deletion. One can speculate on several explanations for these findings. First, H3K79 residue methylated by DOT1L is located near the central nucleosome, unlike H3K9 residue that is located in the histone tail.^40^ This difference may result in H3K79 methylation loss affecting chromatin structure differently in WT mice compared with *Zmpste24*‐deleted mice, which have compromised nuclear membrane. Alternatively, there may be yet undelineated interactions (either direct or indirect) with *Dot1l*/H3K79 methylation and *Zmpste24*/LAMIN. Prior studies show that LAMIN and LAMIN‐binding proteins at the inner nuclear membrane associate with and regulate chromatin conformation,^41^ and that *Dot1l* deletion led to reduced heterochromatic marks in centromeres and telomeres.^42^ Given these results, H3K79 methylation and inner nuclear membrane LAMIN may interact to control euchromatin/heterochromatin structure. Another possibility is confounding cross‐inhibition of other methyltransferases with DOT1L inhibitor EPZ‐5676, but this is less likely given reported 37,000‐fold selectivity of the inhibitor.^43^ Also, there is technical difference between the use of chondrocyte‐specific genetic *Dot1l* deletion and systemic chemical inhibition of DOT1L. The latter affects not only chondrocytes but all cells, including osteoblast, osteocyte, osteoclast, and synovial cells, which may have contributed to differences in phenotype.

In conclusion, *Dot1l* is important in prenatal and postnatal chondrocyte development as well as trabecular bone maintenance. *Dot1l* prevents the premature cell cycle exit of proliferating chondrocytes and supports chondrocyte matrix production. Further studies are necessary to delineate the complex *Dot1l* regulation of chondrocyte–osteoblast/osteocyte homeostasis and its therapeutic potential in human osteoarthritis and osteoporosis.

## Disclosures

Authors have nothing to disclose.

## Supporting information


**Fig S1.** Confirmation of Dot1l knockout mouse model in Dot1lΔ/Δ and Dot1lΔ/Δ; Acan‐CreER mice. (A) Genomic DNA PCR after Cre induction with tamoxife. Chondrocytes were harvested from femoral head (7‐week‐old mice, 4 weeks after injection) and xiphoid process (15‐week‐old mice, 12 weeks after injection) and genomic DNA PCR reaction confirmed Dot1l exon 2 excision. (B) Dot1l qPCR 4 weeks after Cre induction with tamoxifen. Chondrocytes were harvested from femoral head (7‐week‐old mice, 4 weeks after injection) and cDNA qPCR reaction confirmed decreased Dot1l RNA transcript. Residual transcripts may reflect exon 2 excised Dot1l transcript before degradation. Black = control, gray = Dot1l deletion.

**Figure S2.** Figure S2 Ratio of body weight between gender‐matched littermate Dot1lΔ/Δ and Dot1l deleted Dot1lΔ/Δ; Acan‐CreER mice. There is mild decrease in body weight in 15‐week‐old mice, 12 weeks after Dot1l deletion. Data shows representative findings from four litters of male and four litters of female mice (male control n = 4, male Dot1l deletion n = 10, female control n = 7, female Dot1l deletion n = 11). Black = control, gray = Dot1l deletion.

**Figure S3.** Alcian blue staining of L4‐5 disc of Dot1lΔ/Δ and Dot1lΔ/Δ; Acan‐CreER 4‐week‐old mice, 1 week after Dot1l deletion and 15‐week‐old mice, 12 weeks after Dot1l deletion showed hypertrophic zone growth plate disruption and decreased extracellular matrix (n = 3). Scale bar = 100 μm.

**Table S1.** Genotyping primers
**Table S2.** qPCR primers.

## References

[1] Centers for Disease Control . Osteoarthritis (OA) | Basics | Arthritis | CDC. 2018. Available from: https://www.cdc.gov/arthritis/basics/osteoarthritis.htm.

[2] Kotlarz H , Gunnarsson CL , Fang H , Rizzo JA . Insurer and out‐of‐pocket costs of osteoarthritis in the US: evidence from national survey data. Arthritis Rheum. 2009;60(12):3546–53. 19950287 10.1002/art.24984

[3] Christensen R , Henriksen M , Leeds AR , Gudbergsen H , Christensen P , Tina J Sørensen TJ , et al. Effect of weight maintenance on symptoms of knee osteoarthritis in obese patients: a twelve‐month randomized controlled trial. Arthritis Care Res (Hoboken). 2015;67(5):640–50. 25370359 10.1002/acr.22504 PMC4657487

[4] Moyer RF , Hunter DJ . Osteoarthritis in 2014: changing how we define and treat patients with OA. Nat Rev Rheumatol. 2015;11(2):65–6. 25512014 10.1038/nrrheum.2014.211

[5] AbdelSalam H , Restrepo C , Tarity TD , Sangster W , Parvizi J . Predictors of intensive care unit admission after total joint arthroplasty. J Arthroplasty. 2012;27(5):720–5. 22088781 10.1016/j.arth.2011.09.027

[6] Derar H , Shahinpoor M . Recent patents and designs on hip replacement prostheses. Open Biomed Eng J. 2015;9:92–102. 25893020 10.2174/1874120701509010092 PMC4397822

[7] Hamilton DF , Howie CR , Burnett R , Simpson AH , Patton JT . Dealing with the predicted increase in demand for revision total knee arthroplasty: challenges, risks and opportunities. Bone Joint J. 2015;97‐B(6):723–8. 10.1302/0301-620X.97B6.35185 26033049

[8] Centers for Disease Control . FastStats ‐ Osteoporosis. 2017 Available from: https://www.cdc.gov/nchs/fastats/osteoporosis.htm.

[9] Nijmeijer WS , Folbert EC , Vermeer M , Slaets JP , Hegeman JH . Prediction of early mortality following hip fracture surgery in frail elderly: the Almelo hip fracture score (AHFS). Injury. 2016;47(10):2138–43. 27469403 10.1016/j.injury.2016.07.022

[10] Zhou Z , Chen C , Zhang J , Ji X , Liu L , Zhang G , et al., Safety of denosumab in postmenopausal women with osteoporosis or low bone mineral density: a meta‐analysis. Int J Clin Exp Pathol. 2014;7(5):2113–22. 24966919 PMC4069896

[11] Black DM , Rosen CJ . Clinical practice. Postmenopausal osteoporosis. 2016;374(3):254–62. 10.1056/NEJMcp1513724 26789873

[12] van Leeuwen F , Gafken P , Gottschling D . Dot1p modulates silencing in yeast by methylation of the nucleosome core. Cell. 2002;109(6):745–56. 12086673 10.1016/s0092-8674(02)00759-6

[13] Ng HH , Feng Q , Wang H , et al. Lysine methylation within the globular domain of histone H3 by Dot1 is important for telomeric silencing and sir protein association. Genes Dev. 2002;16(12):1518–27. 12080090 10.1101/gad.1001502 PMC186335

[14] Zhou Y , Bi F , Yang G . Association between single nucleotide polymorphisms of DOT1L gene and risk of knee osteoarthritis in a Chinese Han population. Cell Biochem Biophys. 2014;70(3):1677–82. 25005768 10.1007/s12013-014-0112-4

[15] Evangelou E , Valdes AM , Castano‐Betancourt MC , et al. The DOT1L rs12982744 polymorphism is associated with osteoarthritis of the hip with genome‐wide statistical significance in males. Ann Rheum Dis. 2013;72(7):1264–5. 23505243 10.1136/annrheumdis-2012-203182 PMC3686326

[16] Castaño Betancourt MC , Cailotto F , Kerkhof HJ , et al. Genome‐wide association and functional studies identify the DOT1L gene to be involved in cartilage thickness and hip osteoarthritis. Proc Natl Acad Sci U S A. 2012;109(21):8218–23. 22566624 10.1073/pnas.1119899109 PMC3361426

[17] Monteagudo S , FMF C , Aznar‐Lopez C , et al. DOT1L safeguards cartilage homeostasis and protects against osteoarthritis. Nat Commun. 2017;8:15889. 28627522 10.1038/ncomms15889 PMC5481839

[18] Gao Y , Ge W . The histone methyltransferase DOT1L inhibits osteoclastogenesis and protects against osteoporosis. Cell Death Dis. 2018;9(2):33. 29348610 10.1038/s41419-017-0040-5 PMC5833786

[19] F M F Cornelis FMF , de Roover A , Storms L , Hens A , Lories RJ , Monteagudo S . Increased susceptibility to develop spontaneous and post‐traumatic osteoarthritis in Dot1l‐deficient mice. Osteoarthr Cartil. 2018;27(3):513–25. 30513362 10.1016/j.joca.2018.11.008

[20] Jo SY , Granowicz EM , Maillard I , Thomas D , Hess JL . Requirement for Dot1l in murine postnatal hematopoiesis and leukemogenesis by MLL translocation. Blood. 2011;117(18):4759–68. 21398221 10.1182/blood-2010-12-327668 PMC3100687

[21] Ahrens MJ , Romereim S , Dudley AT . A re‐evaluation of two key reagents for in vivo studies of Wnt signaling. Dev Dyn. 2011;240(9):2060–8. 21793100 10.1002/dvdy.22704 PMC3192924

[22] Henry SP , Jang C‐W , Deng JM , Zhang Z , Behringer RR , de Crombrugghe B . Generation of aggrecan‐CreERT2 knockin mice for inducible Cre activity in adult cartilage. Genesis. 2009;47(12):805–14. 19830818 10.1002/dvg.20564 PMC3951921

[23] Domowicz M , Wadlington NL , Henry JG , Diaz K , Munoz MJ , Schwartz NB . Glial cell responses in a murine multifactorial perinatal brain injury model. Brain Res. 2018;1681:52–63. 29274879 10.1016/j.brainres.2017.12.020 PMC5780221

[24] Phornphutkul C , Gruppuso PA . Disorders of the growth plate. Curr Opin Endocrinol Diabetes Obes. 2009;16(6):430–4. 19770649 10.1097/MED.0b013e328331dca2 PMC2894809

[25] Parkinson IH , Fazzalari NL . Interrelationships between structural parameters of cancellous bone reveal accelerated structural change at low bone volume. J Bone Miner Res. 2003;18(12):2200–5. 14672355 10.1359/jbmr.2003.18.12.2200

[26] Kim W , Choi M , Kim JE . The histone methyltransferase Dot1/DOT1L as a critical regulator of the cell cycle. Cell Cycle. 2014;13(5):726–38. 24526115 10.4161/cc.28104 PMC3979909

[27] San‐Segundo PA , Roeder GS . Role for the silencing protein Dot1 in meiotic checkpoint control. Mol Biol Cell. 2000;11(10):3601–15. 11029058 10.1091/mbc.11.10.3601 PMC15018

[28] Ontoso D , Acosta I , van Leeuwen F , Freire R , San‐Segundo PA . Dot1‐dependent histone H3K79 methylation promotes activation of the Mek1 meiotic checkpoint effector kinase by regulating the Hop1 adaptor. PLoS Genet. 2013;9(1):e1003262. 23382701 10.1371/journal.pgen.1003262 PMC3561090

[29] Kang SM , Yoon MH , Park BJ . Laminopathies; mutations on single gene and various human genetic diseases. BMB Rep. 2018;51(7):327–37. 29764566 10.5483/BMBRep.2018.51.7.113 PMC6089866

[30] Osorio FG , Varela I , Lara E , et al. Nuclear envelope alterations generate an aging‐like epigenetic pattern in mice deficient in Zmpste24 metalloprotease. Aging Cell. 2010;9(6):947–57. 20961378 10.1111/j.1474-9726.2010.00621.x

[31] Liu B , Wang Z , Zhang L , Ghosh S , Zheng H , Zhou Z . Depleting the methyltransferase Suv39h1 improves DNA repair and extends lifespan in a progeria mouse model. Nat Commun. 2013;4:1868. 23695662 10.1038/ncomms2885 PMC3674265

[32] Lauing KL , Cortes M , Domowicz MS , Henry JG , Baria AT , Schwartz NB . Aggrecan is required for growth plate cytoarchitecture and differentiation. Dev Biol. 2014;396(2):224–36. 25446537 10.1016/j.ydbio.2014.10.005 PMC4261049

[33] Domowicz MS , Cortes M , Henry JG , Schwartz NB . Aggrecan modulation of growth plate morphogenesis. Dev Biol. 2009;329(2):242–57. 19268444 10.1016/j.ydbio.2009.02.024 PMC2810547

[34] Metsäranta M , Garofalo S , Decker G , Rintala M , de Crombrugghe B , Vuorio E . Chondrodysplasia in transgenic mice harboring a 15‐amino acid deletion in the triple helical domain of pro alpha 1(II) collagen chain. J Cell Biol. 1992;118(1):203–12. 1618904 10.1083/jcb.118.1.203 PMC2289514

[35] Franz H , Villarreal A , Heidrich S , et al. DOT1L promotes progenitor proliferation and primes neuronal layer identity in the developing cerebral cortex. Nucleic Acids Res. 2019;47(1):168–83. 30329130 10.1093/nar/gky953 PMC6326801

[36] Casanova M , Schindeler A , Little D , Müller R , Schneider P . Quantitative phenotyping of bone fracture repair: a review. Bonekey Rep. 2014;3:550. 25120907 10.1038/bonekey.2014.45 PMC4119206

[37] Yang L , Tsang KY , Tang HC , Chan D , Cheah KS . Hypertrophic chondrocytes can become osteoblasts and osteocytes in endochondral bone formation. Proc Natl Acad Sci U S A. 2014;111(33):12097–102. 25092332 10.1073/pnas.1302703111 PMC4143064

[38] Zhou X , von der Mark K , Henry S , Norton W , Adams H , de Crombrugghe B . Chondrocytes transdifferentiate into osteoblasts in endochondral bone during development, postnatal growth and fracture healing in mice. PLoS Genet. 2014;10(12):e1004820. 25474590 10.1371/journal.pgen.1004820 PMC4256265

[39] Savic D , Pedoia V , Seo Y , et al. Imaging bone‐cartilage interactions in osteoarthritis using [^18^F]‐NaF PET‐MRI. Mol Imaging. 2016;15:1–12. 28654417 10.1177/1536012116683597 PMC5470142

[40] Kouzarides T . Chromatin modifications and their function. Cell. 2007;128(4):693–705. 17320507 10.1016/j.cell.2007.02.005

[41] Naetar N , Ferraioli S , Foisner R . Lamins in the nuclear interior ‐ life outside the lamina. J Cell Sci. 2017;130(13):2087–96. 28668931 10.1242/jcs.203430

[42] Jones B , Su H , Bhat A , et al. The histone H3K79 methyltransferase Dot1L is essential for mammalian development and heterochromatin structure. PLoS Genet. 2008;4(9):e1000190. 18787701 10.1371/journal.pgen.1000190 PMC2527135

[43] Daigle SR , Olhava EJ , Therkelsen CA , et al. Potent inhibition of DOT1L as treatment of MLL‐fusion leukemia. Blood. 2013;122(6):1017–25. 23801631 10.1182/blood-2013-04-497644 PMC3739029

