# Supplementary figures and images for: The Role of Dot1l in Prenatal and Postnatal Murine Chondrocytes and Trabecular Bone
Source: JBMR Plus. 2019 Dec 17;4(2):e10254. doi: 10.1002/jbm4.10254 (PMC7017886; doi:10.1002/jbm4.10254)

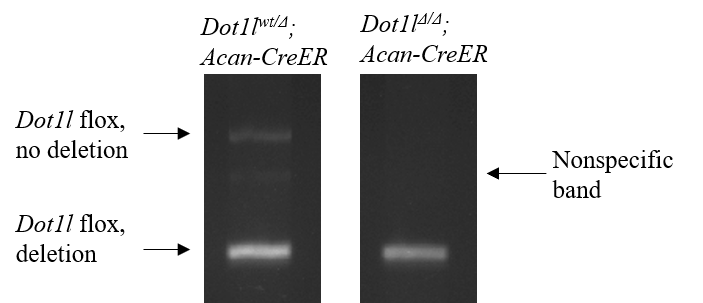

Supplement: Supplementary file 1 — Fig S1. Confirmation of Dot1l knockout mouse model in Dot1lΔ/Δ and Dot1lΔ/Δ; Acan‐CreER mice. (A) Genomic DNA PCR after Cre induction with tamoxife. Chondrocytes were harvested from femoral head (7‐week‐old mice, 4 weeks after injection) and xiphoid process (15‐week‐old mice, 12 weeks after injection) and genomic DNA PCR reaction confirmed Dot1l exon 2 excision. (B) Dot1l qPCR 4 weeks after Cre induction with tamoxifen. Chondrocytes were harvested from femoral head (7‐week‐old mice, 4 weeks after injection) and cDNA qPCR reaction confirmed decreased Dot1l RNA transcript. Residual transcripts may reflect exon 2 excised Dot1l transcript before degradation. Black = control, gray = Dot1l deletion. [file JBM4-4-e10254-s001.zip › JBM4_10254_S1A.tiff]

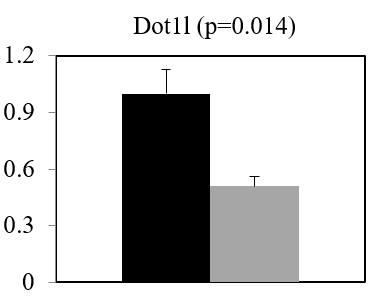

Supplement: Supplementary file 1 — Fig S1. Confirmation of Dot1l knockout mouse model in Dot1lΔ/Δ and Dot1lΔ/Δ; Acan‐CreER mice. (A) Genomic DNA PCR after Cre induction with tamoxife. Chondrocytes were harvested from femoral head (7‐week‐old mice, 4 weeks after injection) and xiphoid process (15‐week‐old mice, 12 weeks after injection) and genomic DNA PCR reaction confirmed Dot1l exon 2 excision. (B) Dot1l qPCR 4 weeks after Cre induction with tamoxifen. Chondrocytes were harvested from femoral head (7‐week‐old mice, 4 weeks after injection) and cDNA qPCR reaction confirmed decreased Dot1l RNA transcript. Residual transcripts may reflect exon 2 excised Dot1l transcript before degradation. Black = control, gray = Dot1l deletion. [file JBM4-4-e10254-s001.zip › JBM4_10254_S1B.tiff]

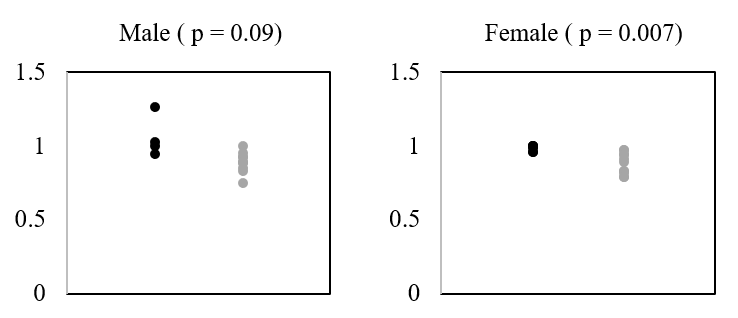

Supplement: Supplementary file 2 — Figure S2. Figure S2 Ratio of body weight between gender‐matched littermate Dot1lΔ/Δ and Dot1l deleted Dot1lΔ/Δ; Acan‐CreER mice. There is mild decrease in body weight in 15‐week‐old mice, 12 weeks after Dot1l deletion. Data shows representative findings from four litters of male and four litters of female mice (male control n = 4, male Dot1l deletion n = 10, female control n = 7, female Dot1l deletion n = 11). Black = control, gray = Dot1l deletion. [file JBM4-4-e10254-s002.tiff]

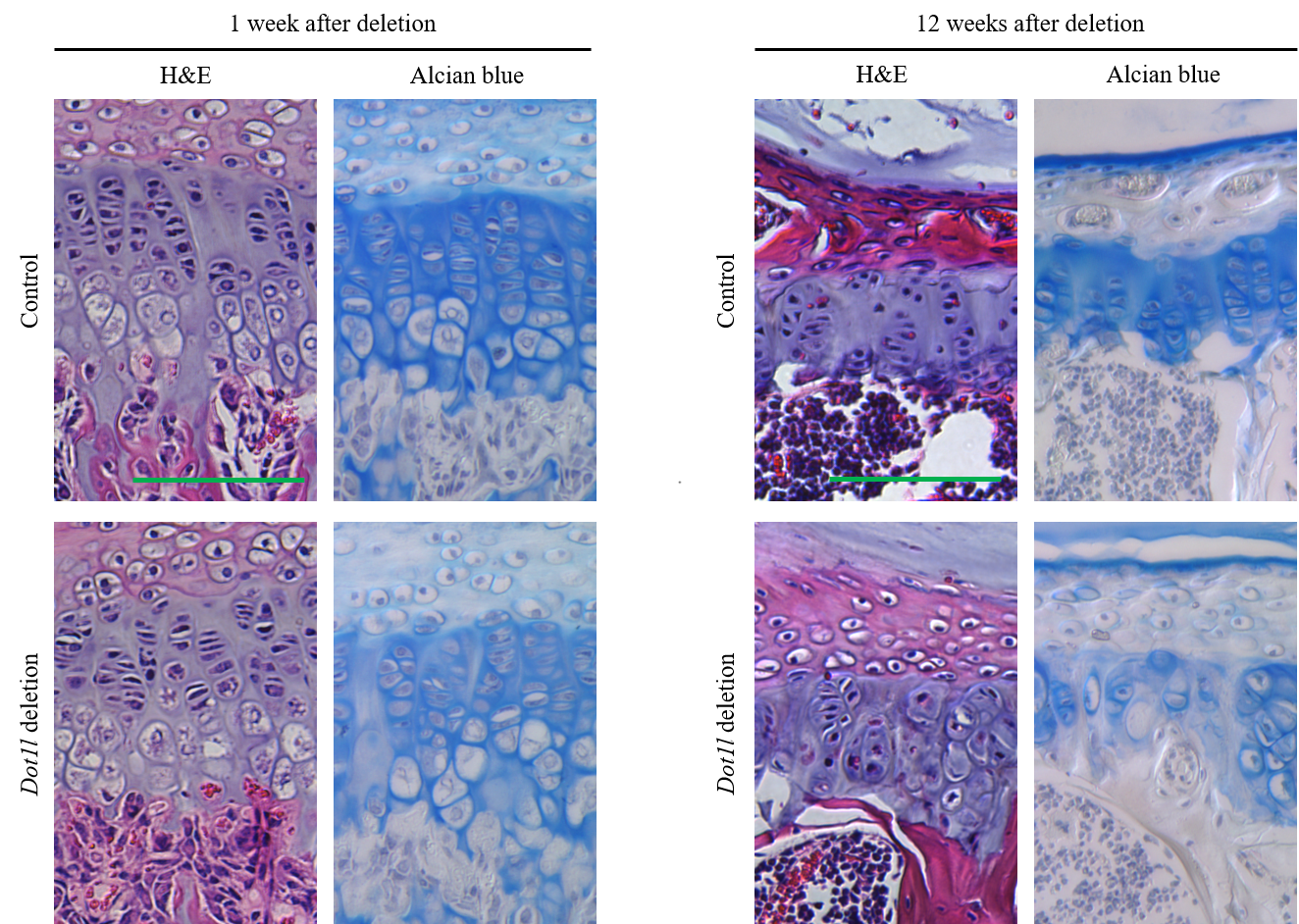

Supplement: Supplementary file 3 — Figure S3. Alcian blue staining of L4‐5 disc of Dot1lΔ/Δ and Dot1lΔ/Δ; Acan‐CreER 4‐week‐old mice, 1 week after Dot1l deletion and 15‐week‐old mice, 12 weeks after Dot1l deletion showed hypertrophic zone growth plate disruption and decreased extracellular matrix (n = 3). Scale bar = 100 μm. [file JBM4-4-e10254-s003.tiff]
