# Supplementary material for: The Role of Dot1l in Prenatal and Postnatal Murine Chondrocytes and Trabecular Bone
Source: JBMR Plus. 2019 Dec 17;4(2):e10254. doi: 10.1002/jbm4.10254 (PMC7017886; doi:10.1002/jbm4.10254)
Supplement: Supplementary file 4 — Table S1. Genotyping primers Table S2. qPCR primers. [file JBM4-4-e10254-s004.docx]

Table S1. Genotyping primers

| **Allele** | **Sequences** |
| --- | --- |
| loxp flanked *Dot1l* allele | F: GCCTACAGCCTTCATCATTC  R: GATAGTCTCAATAATCTCA |
| *Dot1l* excised allele | F: GAAGTTCCTATTCCGAAGTT  R: GAACCACAGGATGCTTCAG |
| *Acan-CreER* | F: GTTATATTCCGGAGC CCACA  Mid: AAAAGCGACAAGAAGACACCA  R: CTCCAGACTGCCTTGGGAAAA |
| *Cre* | F: GCATTACCGGTCGATGCAACGAGTGATGAG  R: GAGTGAACGAACCTGGTCGAAATCAGTGCG |

Table S2. qPCR primers

| **Gene** | **Forward** | **Reverse** |
| --- | --- | --- |
| *Actb* (control) | GTTGGAGCAAACATCCCCCA | CGCGACCATCCTCCTCTTAG |
| *Dot1l* | GCCAGCTTGAGATCTGGGATT | GCTGCCGGTCTACGACAAG |
| *Acan* | ACCCGGTACCCTACAGAGAC | GTCCACCCCTCCTCACATTG |
| *Col2a1* | GTTTGCCAGCCTTTGGAGCG | TTTCTGCCCCTTTGGCCCTA |
